# ASD and Perceptual Disturbances - Do People With ASD Have an Increased Risk of Visual and Auditory Hallucinations?

**DOI:** 10.1192/bjo.2025.10263

**Published:** 2025-06-20

**Authors:** Dawn Collins, Jo Lowe

**Affiliations:** 1 NSFT, Great Yarmouth, United Kingdom; 2 NSFT, Norwich, United Kingdom

## Abstract

**Aims:** To establish if there is data to support the clinical impression that people with ASD are more likely to experience perceptual disturbances (visual and auditory).

**Methods:** Literature search using Athens/Pub Med. Clinical observations had been that young people with ASD seemed to experience increased perceptual disturbances but that these did not respond to antipsychotics. Detailed history taking also suggested that, especially visual hallucinations, were often long-standing and had started in mid childhood (typically while at primary school). These tended not to cause distress initially but often increased during adolescence.

**Results:** There is little specific data on this subject: there are numerous studies and case reports considering the increased risk of psychosis and schizophrenia in people with ASD but not specifically on non-psychotic young people with ASD who have perceptual disturbances (in the absence of other symptoms suggesting psychosis). Limited data that was available noted that people with ASD were 3 times more likely to experience auditory and/or visual hallucinations than their counterparts. Suggested pathways for this included shared pathological pathways (between schizophrenia and ASD), overlapping DNA (not established), that ASD is a risk factor for later development of schizophrenia and living with ASD may incur increased social stressors (bullying, exclusion, marginalisation, isolation etc.).

**Conclusion:** Having ASD does appear to increase the likelihood of experiencing (psychotic and non-psychotic) perceptual disturbances. The reasons for this are largely inconclusive but may explain our clinical impression; that young people with ASD who are hearing voices or seeing things but who are not psychotic, do not appear to respond positively to antipsychotics. We would advise medications are used with caution in this (non-psychotic) patient group and that other avenues are considered for treatment, including self-help, psychoeducation and psychological support. We must be cautious about causing iatrogenic harm and over medicating. More research is needed in this field.

